# Identification of a novel nidovirus in an outbreak of fatal respiratory disease in ball pythons (*Python regius*)

**DOI:** 10.1186/1743-422X-11-144

**Published:** 2014-08-08

**Authors:** Lorenzo Uccellini, Robert J Ossiboff, Ricardo EC de Matos, James K Morrisey, Alexandra Petrosov, Isamara Navarrete-Macias, Komal Jain, Allison L Hicks, Elizabeth L Buckles, Rafal Tokarz, Denise McAloose, Walter Ian Lipkin

**Affiliations:** Center for Infection and Immunity, Mailman School of Public Health, Columbia University, New York, USA; Department of Biomedical Sciences, Cornell University, Ithaca, NY 14853 USA; Wildlife Conservation Society, Zoological Health Program, Bronx, NY 10460 USA; Department of Clinical Sciences, College of Veterinary Medicine, Cornell University, Ithaca, NY 14853 USA

**Keywords:** *Nidovirales*, Pneumonia, Reptile, Snake

## Abstract

**Background:**

Respiratory infections are important causes of morbidity and mortality in reptiles; however, the causative agents are only infrequently identified.

**Findings:**

Pneumonia, tracheitis and esophagitis were reported in a collection of ball pythons (*Python regius*). Eight of 12 snakes had evidence of bacterial pneumonia. High-throughput sequencing of total extracted nucleic acids from lung, esophagus and spleen revealed a novel nidovirus. PCR indicated the presence of viral RNA in lung, trachea, esophagus, liver, and spleen. In situ hybridization confirmed the presence of intracellular, intracytoplasmic viral nucleic acids in the lungs of infected snakes. Phylogenetic analysis based on a 1,136 amino acid segment of the polyprotein suggests that this virus may represent a new species in the subfamily *Torovirinae.*

**Conclusions:**

This report of a novel nidovirus in ball pythons may provide insight into the pathogenesis of respiratory disease in this species and enhances our knowledge of the diversity of nidoviruses.

## Findings

*Nidovirales* is a large order of positive sense, single-stranded RNA (ssRNA) viruses that consists of the many genera and species in the families *Coronaviridae*, *Arteriviridae*, *Roniviridae* and *Mesoniviridae*. Although the genomes of nidoviruses vary in length, ranging from 13 to 32 kilobases (kb), the organization of the genomes are similar across the entire order [1–4]. The 5′ end of the genome encodes two replicase polyproteins (pp1a and pp1ab), structural proteins and accessory proteins. Genes downstream of the replicase polyprotein gene are expressed from a nested set of 3′-coterminal subgenomic mRNAs, a replication strategy unique to the *Nidovirales* [5–7].

Nidoviruses infect a broad range of hosts including humans and other mammals, birds, fish, insects and crustaceans [8–11]. Although reptiles are susceptible to infection by a wide variety of viruses (as reviewed in [12]), nidovirus infections have not previously been described. Viruses affecting the reptile respiratory tract include herpesviruses [13], iridoviruses [14], adenoviruses [15], flaviviruses [16], and, of particular importance in snakes, paramyxoviruses [17] and reoviruses [18].

Here we report the discovery of a novel nidovirus in a collection of ball pythons (*Python regius*) in upstate New York with pneumonia, tracheitis and esophagitis. The snakes were found dead between July 2011 and September 2013.

Gross postmortem examination was performed on 4 snakes. Snake 1, a 6-year-old, female piebald color morph ball python, was submitted in July of 2011. Snake 2, a 6-year-old, male lesser platinum color morph ball python, was submitted in December of 2011. Snake 3, a 3-year-old female paradox color morph ball python, and snake 4, a 7-year-old female pastel color morph ball python, were submitted in September 2013. After gross evaluation, samples of tissues were collected and saved in 10% neutral buffered formalin, routinely processed and mounted in paraffin. Five μm paraffin ribbons were cut and stained with either hematoxylin and eosin (H&E) or Gram’s stain for histologic examination. Eleven tissues, including lung (n = 4), liver (n = 1), spleen (n = 3) and esophagus (n = 3) from the 4 snakes were collected and stored at −20°C.

**Figure 1:**
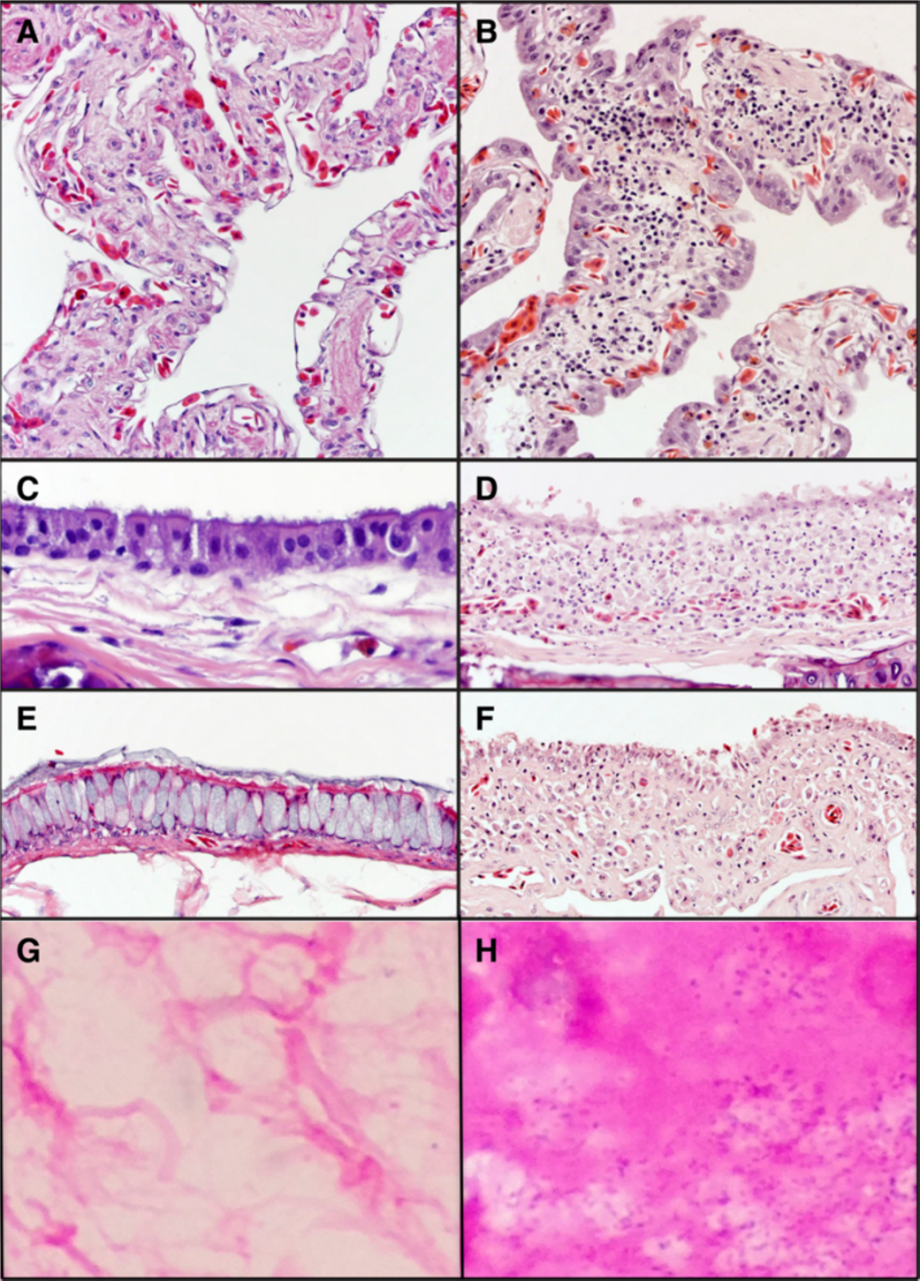
**Histologic pulmonary, tracheal and esophageal lesions in infected ball pythons. (A-F)**: hematoxylin and eosin staining, magnification 400-600X. **(G,H)**: Gram-staining, magnification 1000X. **(A,C,E)**: histology of uninfected snake; **(B,D,F)**: histology of infected snakes. The lungs from infected snakes **(B)** were characterized by marked pneumocyte hyperplasia and mixed mononuclear and granulocytic inflammation compared to the uninfected snake **(A)**. The normally thin and ciliated tracheal mucosa **(C)** was severely thickened in infected snakes **(D)** with epithelial necrosis, loss of the ciliated mucosal border and moderate mixed inflammation. The esophageal mucosa that is normally rich with mucus-producing epithelial cells **(E)** was also severely hyperplastic in infected snakes, with necrosis and mixed inflammatory infiltrates similar to those seen in the trachea **(F)**. Gram-negative bacteria are present in snake with bronchopneumonia **(H)** but not in snake without bronchopneumonia **(G)**.

Gross and histologic findings in all four snakes were primarily restricted to the respiratory and upper gastrointestinal tracts (Table 1). Hematoxylin and eosin stained sections (Figure 1A through F) revealed marked hyperplasia of epithelial cells lining air exchange areas (pneumocytes) with significant mononuclear (lymphocytes and plasma cells) and granulocytic (heterophils) interstitial inflammation and epithelial necrosis (Figure 1B). Similar inflammatory and hyperplastic changes were also present in the trachea (Figure 1D), esophagus (Figure 1F) and oral cavity. Gram-negative stained bacteria are shown in lung tissue from a snake with bacterial bronchopneumonia (Figure 1H).

**Table 1:** **Pathologic and molecular findings in ball pythons (** ***Python regius*** **) with nidoviral-associated disease**

Inflammation ^a^									PCR ^b^					ISH ^c^
Snake	Lung	Trachea	Nasal Cavity	Esophagus	Oral Cavity	Liver	Spleen	Kidney	Lung	Spleen	Esophagus	Trachea	Liver	Lung
1	+, H	+, H	+	+, H	+	-	-	-	+	+	NT	NT	NT	+
2	+	+	+	+, H	+	Nec	-	-	+	+	+	NT	NT	+
3	+	+	NE	NE	+	-	Nec	-	NA	+	NA	NT	+	NT
4	+	+	NE	NE	+	-	NE	-	+	NT	NT	+	NT	NT

^a^H, epithelial hyperplasia; NE, Not examined; Nec, Necrosis.

^b^NT, Not tested.

^c^ISH, In Situ Hybridization; NT, Not tested NA, not available.

+, PCR Positive.

-, PCR Negative.

Total nucleic acids were extracted from snake samples (lung and spleen for snake 1, lung, spleen and esophagus for snake 2, spleen and liver for snake 3, lung and trachea for snake 4) using the EasyMag (bioMérieux, Inc.) platform; Samples from snakes 1 and 2 were depleted of ribosomal RNA (Ribo-Zero™ rRNA Removal, Epibio) and treated with DNAse I (TURBO DNA-free™, Ambion). cDNA synthesis was performed using SuperScript II first-strand synthesis supermix (Invitrogen). Viral discovery was performed using broadly reactive consensus PCR assays targeting common respiratory viruses of animals, including paramyxoviruses [19–21], reoviruses [16] and caliciviruses [22]. When PCR analysis failed to yield a causative agent, high-throughput sequencing was performed on all samples originating from snakes 1 and 2 (Ion PGM, Life Sciences). On average, 850,000 reads were obtained from each sample. All reads were processed by trimming primers and adaptors, length filtering, and masking of low-complexity regions (WU-BLAST 2.0). To remove host sequences, the remaining reads were subjected to a homology search using BLASTn against a database consisting of ribosomal and genomic metazoan sequences. Following the processing, an average of 250,000 reads per sample remained for further analysis.

Nucleotide sequence analysis (BLASTn) of processed reads was uninformative; however, amino acid analysis (BLASTx) revealed multiple reads with amino acid homology of <50% to the polyprotein region of the *Nidovirales* subfamily *Torovirinae*, including Breda virus, White bream virus, and Fathead minnow virus. Assembly of all *Torovirinae*-like reads generated a 3,408 nt contig with 33% amino acid homology to the replicase polyprotein 1ab of Fathead minnow virus. The presence of this 3,408 nt sequence in both snakes was confirmed by PCR using primers shown in Table 2. Cycling conditions are described in a footnote to Table 2. Samples from multiple tissues of snakes 3 and 4 were also screened and tested positive for this virus.

**Table 2:** **Sequences of primers used to amplify the 3,408 fragment of ball python Nidovirus**

Primer name*	Sequence 5′ – 3′	Nucleotide position
BPNV 1Fwd BPNV 1Rev	ACCTGCTACCGATGTCCAAG GTCGTTGTTGGCTGAGTGTG	1-963
BPNV 2Fwd BPNV 2Rev	TTCAAGCGAACCAAGTTCATCC TCTTGGACATCGGTAGCAGG	718-1485
BPNV 3Fwd BPNV 3Rev	AACATCCTCGACAACGCAGG ACGTAGTCTTGCCAGTTCCC	1446-1647
BPNV 4Fwd BPNV 4Rev	CCACAACCCGACAGTCAGTA GTACGTAGTCTTGCCAGTTCC	1456-2154
BPNV 5Fwd BPNV 5Rev	GGCACAGTAACAGCACAACG GTACTGCAAGATGCCGTTGC	2152-3030
BPNV 6Fwd BPNV 6Rev	GTGACTACACGAAATGCGACC GTCAAACATGAAAGCGTGCG	2995-3131
BPNV 7Fwd BPNV 7Rev	GTCGTCAACTTGTCCCACCA CTGCCATGCTACGGAAGACT	3088-3408

*PCR cycling conditions: 95°C for 10 minutes followed by 35 cycles of 95**°**C for 30 seconds, 55°C for 30 seconds, 72°C for 1 minute, except primer pair BPNV 1 and BPNV 6 where annealing temperature was 53°C.

**Figure 2:**
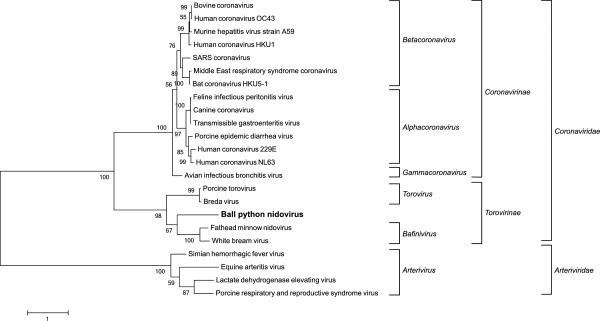
**Phylogenetic tree based on 1,136 amino acid segment of the polyprotein the novel ball python nidovirus.** A bootstrap-supported (1000 replicates) Maximum Likelihood tree was implemented in MEGA version 5.2. Unrooted tree is shown with bootstrap values at the nodes. Scale bars represent 0.5 amino acid substitutions per site. GenBank accession number: Bovine coronavirus: NC003045, Human coronavirus OC43:NC005147, Murine hepatitis virus strain A59:NC001846, Human coronavirus HKU1:NC006577, SARS coronavirus: NC004718, Middle East respiratory syndrome coronavirus: NC019843, Bat coronavirus HKU5-1:NC009020, Feline infectious peritonitis virus: NC002306, Canine coronavirus strain S378:KC175341, Transmissible gastroenteritis virus isolate TGEV-HX:KC962433, Porcine epidemic diarrhea virus: NC003436, Human coronavirus 229E:NC002645, Human coronavirus NL63:NC005831, Avian infectious bronchitis virus: NC001451, Fathead minnow nidovirus: GU002364, White bream virus: NC008516, Porcine torovirus strain SH1:JQ860350, Breda Virus:NC007447, Lactate dehydrogenase elevating virus: NC001639, Porcine respiratory and reproductive syndrome virus: NC001961, Simian hemorrhagic fever virus: NC003092, Equine arteritis virus: NC002532.

**Figure 3:**
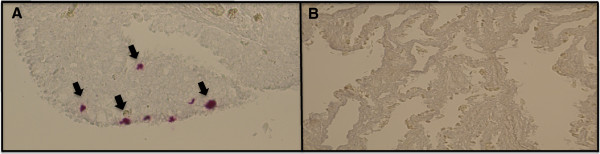
**Colocalization of BPNV nucleic acid and pulmonary lesions by in situ hybridization. (A)** Infected lung from Snake 1. Arrows indicate probe bound to virus. Magnification 20x. **(B)** Uninfected, control ball python lung.

For phylogenetic analysis, the 3,408 nt sequence was translated with Se-Al v2.0a11, and a 1,136 amino acid fragment was aligned against all *Nidovirales* sequences from GenBank using ClustalW. The best-fit model of amino acid substitution, the Whelan and Goldman (WAG) matrix, was selected using the maximum likelihood method implemented in MEGA version 5.2 [23]. A Bootstrap-supported (1000 replicates) maximum likelihood phylogenetic tree was constructed using MEGA version 5.2. The ball python-associated virus clustered within the *Torovirinae* subfamily (Figure 2). A neighbor-joining phylogenetic method was also implemented with congruent results. Based on the phylogenetic position and the genetic distances between species, this virus, tentatively called ball python nidovirus (BPNV, GenBank accession number KM267236) may represent a new species within the subfamily *Torovirinae.*In situ hybridization to a 934 nt fragment of the genomic polyprotein 1ab region was used to assess viral infection and distribution in the lung tissue. Positive cytoplasmic staining, consistent with the presence of viral nucleic acid, was confirmed in the cytoplasm of pulmonary cells, presumably epithelial cells (Figure 3A). The specificity of probes for in situ hybridization was confirmed by the absence of signal when the same probe was used on control pulmonary tissue from an uninfected, 6-year-old, female ball python maintained at College of Veterinary Medicine, Cornell University (Figure 3B).

Respiratory disease can be an important cause of morbidity and mortality in both wild and captive reptiles. In captivity, reptiles, and particularly snakes, are frequently maintained in collections with a high population density in relatively small spaces. As such, disease transmission within collections can occur rapidly, and early detection and diagnosis is critical in controlling disease spread.

Although 8 of 12 snakes with disease in the collection showed gram-negative rods by Gram stain and follow up culture in 4 revealed the presence of *Aeromonas sp., Pseudomonas sp., Serratia sp.,* no evidence of bacterial infection was found in 4 snakes. In contrast, all snakes with epithelial hyperplasia in the trachea, lung and esophagus and mononuclear inflammatory infiltrates had viral signal by PCR and ISH. In concert, these data suggest a role for this nidovirus in the pathogenesis of respiratory disease. However, unequivocal implication will require experimental infection studies.

The identification of this novel nidovirus expands our understanding of nidoviral diversity and provides insight into the pathogenesis of respiratory disease in snakes. Phylogenetic analysis indicated that the virus belongs to a novel genus within the *Torovirinae* subfamily distinct from the *Torovirus* and recently characterized *Banifivirus* genera [24]. Due to overlapping clinical signs and pathologic lesions of the newly discovered nidovirus with the best characterized viral respiratory pathogens of snakes, paramyxoviruses and reoviruses [17, 18], it is possible that nidoviral infections were previously misdiagnosed or overlooked. PCR-based detection methods to rapidly determine infection status and etiology of respiratory disease in snakes are recommended to guide decisions for managing husbandry and veterinary care.

## Availability of supporting data

The data set supporting the results of this article is included within the article.

## Abbreviations

ssRNA: Single-stranded RNA
kb: Kilobases
H&E: Hematoxylin and eosin
PCR: Polymerase chain reaction
nt: Nucleotide.

## References

[1] Cavanagh D (1997). Nidovirales: a new order comprising Coronaviridae and Arteriviridae. Arch Virol.

[2] Cowley JA, Dimmock CM, Spann KM, Walker PJ (2000). Gill-associated virus of Penaeus monodon prawns: an invertebrate virus with ORF1a and ORF1b genes related to arteri- and coronaviruses. J Gen Virol.

[3] Lauber C, Ziebuhr J, Junglen S, Drosten C, Zirkel F, Nga PT, Morita K, Snijder EJ, Gorbalenya AE (2012). Mesoniviridae: a proposed new family in the order Nidovirales formed by a single species of mosquito-borne viruses. Arch Virol.

[4] Snijder EJ, Horzinek MC, Spaan WJ (1993). The coronaviruslike superfamily. Adv Exp Med Biol.

[5] Cowley JA, Dimmock CM, Walker PJ (2002). Gill-associated nidovirus of Penaeus monodon prawns transcribes 3-coterminal subgenomic mRNAs that do not possess 5-leader sequences. J Gen Virol.

[6] Pasternak AO, Spaan WJ, Snijder EJ (2006). Nidovirus transcription: how to make sense…?. J Gen Virol.

[7] Sittidilokratna N, Dangtip S, Cowley JA, Walker PJ (2008). RNA transcription analysis and completion of the genome sequence of yellow head nidovirus. Virus Res.

[8] Walker PJ, Bonami JR, Boonsaeng V, Chang PS, Cowley JA, Enjuanes L, Flegel TW, Lightner DV, Loh PC, Snijder EJ, Tang K, Fauquet CM, Mayo MA, Maniloff J, Desselberger U, Ball LA (2005). Virus Taxonomy: Classification and Nomenclature of Viruses. Eighth Report of the International Committee for Taxonomy of Viruses.

[9] Siddell S, Snijder EJ, Perlman S, Gallagher T, Snijder EJ (2008). An Introduction to Nidoviruses. Nidoviruses.

[10] Gaedke K, Zurbriggen A, Baumgärtner W (1997). In vivo and in vitro detection of canine distemper virus nucleoprotein gene with digoxigenin-labelled RNA, double-stranded DNA probes and oligonucleotides by in situ hybridization. Zentralblatt fur Veterinarmedizin Reihe B J Vet Med.

[11] Zirkel F, Kurth A, Quan PL, Briese T, Ellerbrok H, Pauli G, Leendertz FH, Lipkin WI, Ziebuhr J, Drosten C, Junglen S (2011). An insect nidovirus emerging from a primary tropical rainforest. mBio.

[12] Marschang RE (2011). Viruses Infecting Reptiles. Viruses.

[13] Jacobson ER, Gaskin JM, Roelke M, Greiner EC, Allen J (1986). Conjunctivitis, tracheitis, and pneumonia associated with herpesvirus infection in green sea turtles. J Am Vet Med Assoc.

[14] Westhouse RA, Jacobson ER, Harris RK, Winter KR, Homer BL (1996). Respiratory and pharyngo-esophageal iridovirus infection in a gopher tortoise (Gopherus polyphemus). J Wildl Dis.

[15] Jacobson ER, Gardiner CH (1990). Adeno-like virus in esophageal and tracheal mucosa of a Jackson’s chameleon (Chamaeleo jacksoni). Vet Pathol.

[16] Jacobson ER (2007). From Viruses and Viral Diseases of Reptiles. Infectious Diseases and Pathology in Reptiles: Color Atlas and Text.

[17] Jacobson ER, Adams HP, Geisbert TW, Tucker SJ, Hall BJ, Homer BL (1997). Pulmonary lesions in experimental ophidian paramyxovirus pneumonia of Aruba Island rattlesnakes, Crotalus unicolor. Vet Pathol.

[18] Lamirande EW, Nichols DK, Owens JW, Gaskin JM, Jacobson ER (1999). Isolation and experimental transmission of a reovirus pathogenic in ratsnakes (Elaphe species). Virus Res.

[19] Homer BL, Sundberg JP, Gaskin JM, Schumacher J, Jacobson ER (1995). Immunoperoxidase detection of ophidian paramyxovirus in snake lung using a polyclonal antibody. J Vet Diagn Invest.

[20] Orós J, Sicilia J, Torrent A, Castro P, Déniz S, Arencibia A, Jacobson ER, Homer BL (2001). Immunohistochemical detection of ophidian paramyxovirus in snakes in the Canary Islands. Vet Rec.

[21] Sand MA, Latimer KS, Gregory CR, Rakich PM, Jacobson ER, Pennick KE (2004). Molecular diagnosis of paramyxovirus infection in snakes using reverse transcriptase-polymerase chain reaction and complementary deoxyribonucleic acid:ribonucleic acid in situ hybridisation. J Vet Diagn Invest.

[22] Radford AD, Coyne KP, Dawson S, Porter CJ, Gaskell RM (2007). Feline calicivirus. Vet Res.

[23] Tamura K, Peterson D, Peterson N, Stecher G, Nei M, Kumar S (2011). MEGA5: molecular evolutionary genetics analysis using maximum likelihood, evolutionary distance, and maximum parsimony methods. Mol Biol Evol.

[24] Batts WN, Goodwin AE, Winton JR (2012). Genetic analysis of a novel nidovirus from fathead minnows. J Gen Virol.

